# From street level to science: advancing methods for climate walks to improve human thermal comfort

**DOI:** 10.1007/s00484-026-03167-8

**Published:** 2026-04-02

**Authors:** Kevin Lau, Cho Kwong Charlie Lam, Eduardo Krüger, André Santos Nouri, Zhikai Peng, Daniele Santucci, Andreas Matzarakis

**Affiliations:** 1 Department of Civil Engineering and Natural Resources Engineering, Luleå University of Technology, Luleå, Sweden; 2 School of Geography, Earth and Environmental Sciences, University of Plymouth, Plymouth, UK; 3 Department of Civil Construction, Federal University of Technology of Parana, Curitiba, Brazil; 4 Department of Environmental Sciences and Engineering, NOVA School of Science and Technology—NOVA FCT, NOVA University Lisbon—UNL, Campus de Caparica ‑ 2829–516, Caparica, Portugal; 5 MARE – Marine and Environmental Sciences Centre / Associate Laboratory ARNET – Aquatic Research Network, Caparica, Portugal; 6 Department of Architectural Engineering and Technology, Delft University of Technology, Delft, The Netherlands; 7 Climateflux GmbH, Munich, Germany; 8 Chair for Building Technology, RWTH Aachen University, Aachen, Germany; 9 Environmental Meteorology, University of Freiburg, Freiburg im Breisgau, Germany; 10 Democritus University of Thrace, Komotini, Greece

**Keywords:** Mobile environmental monitoring, Urban microclimate, Human thermal comfort, Dynamic conditions

## Abstract

Urban heat risk is increasing, while fixed monitoring networks remain too sparse and coarse to resolve the pedestrian-scale variability, especially radiative loads, that governs outdoor thermal stress. This short communication advances the concept of climate walks, defined as route-based, human-centred field campaigns that build on earlier work on “thermal walks”, and presents them as a practice-ready methodology for design-relevant evidence. We define climate walks as structured, route-based, georeferenced assessments that pair high-resolution mobile microclimate measurements with synchronous in-situ human responses to capture transient, spatially heterogeneous conditions along actual walks. We synthesize key methodological features, such as dynamic, stop-and-go protocols; human-centred sensing; multisensory extensions; accessible kits from research-grade to low-cost platforms; and emerging diagnostics, and show how these produce actionable design measures. We discuss limitations and challenges, including lags and thermal memory, instrumentation and, index choice under transients, and the need for protocol harmonization. We then propose a research agenda to investigate dynamic conditions of outdoor thermal comfort, develop time-resolved, memory-aware comfort metrics, test indices under motion, mainstream multisensory models, and shift practice from isolated cool spots to connected, route-scale cool sequences. Together, these steps link biometeorology to actionable urban planning and design for heat-resilient, attractive public spaces.

**Supplementary Information:**

The online version contains supplementary material available at 10.1007/s00484-026-03167-8.

## Introduction

Cities face increasing risks of extreme heat under climate change, yet most monitoring infrastructures are sparse, fixed, and too coarse to resolve the micro-scale variability that governs pedestrian exposure, especially radiative loads that fluctuate sharply across streets, courtyards, tree rows, and arcades. This limits timely, evidence-based urban planning and design. Recent work in human biometeorology underscores the need to raise the spatial granularity of assessments from station footprints to the pedestrian scale (Nazarian and Lee 2021), arguing that intra-urban differences linked to form, shading, and materials require high-resolution observation to inform adaptation and mitigation measures.

A growing toolbox of microclimatic measurements and monitoring (fixed, mobile, and hybrid) now enables such design-relevant evidence. Non-motorised mobile campaigns and trolley/backpack systems measure air temperature, humidity, wind, mean radiant temperature (T_mrt_; or its components), sky view factor, surface temperatures, and air quality at walking pace (Krüger et al. 2024; Lau et al. 2019; Requena-Ruiz et al. 2025), producing georeferenced datasets that explain how morphology and materials shape thermal conditions at specific places and times. These campaigns have shown that mobile measurements can capture fine-scale contrasts that stations miss and translate them into actionable diagnostics for public-space design. Examples include identifying thermal diversity and shade-driven differences in thermal indices such as physiologically equivalent temperature (PET) (Mayer and Höppe, 1987) or universal thermal climate index (UTCI) (Jendritzky et al., 2012) across positions (Lam et al. 2024) and decomposing radiative drivers (Siret et al. 2025), as well as quantifying the cooling efficacy of interventions such as canopies, trees, misters, and paving changes (Ouyang et al. 2025). Together, these approaches reveal where and when to prioritize shading, ventilation paths, or material choices to improve thermal performance and comfort (Abuwaer et al. 2025).

In this short communication, we (i) articulate the above definition in light of recent methodological advances; (ii) summarize core characteristics and good practice for instrumentation and protocol design; and (iii) motivate a research agenda on dynamic outdoor thermal comfort that can better inform heat-resilient urban planning and design.

## Definition of climate walks

**Fig. 1:**
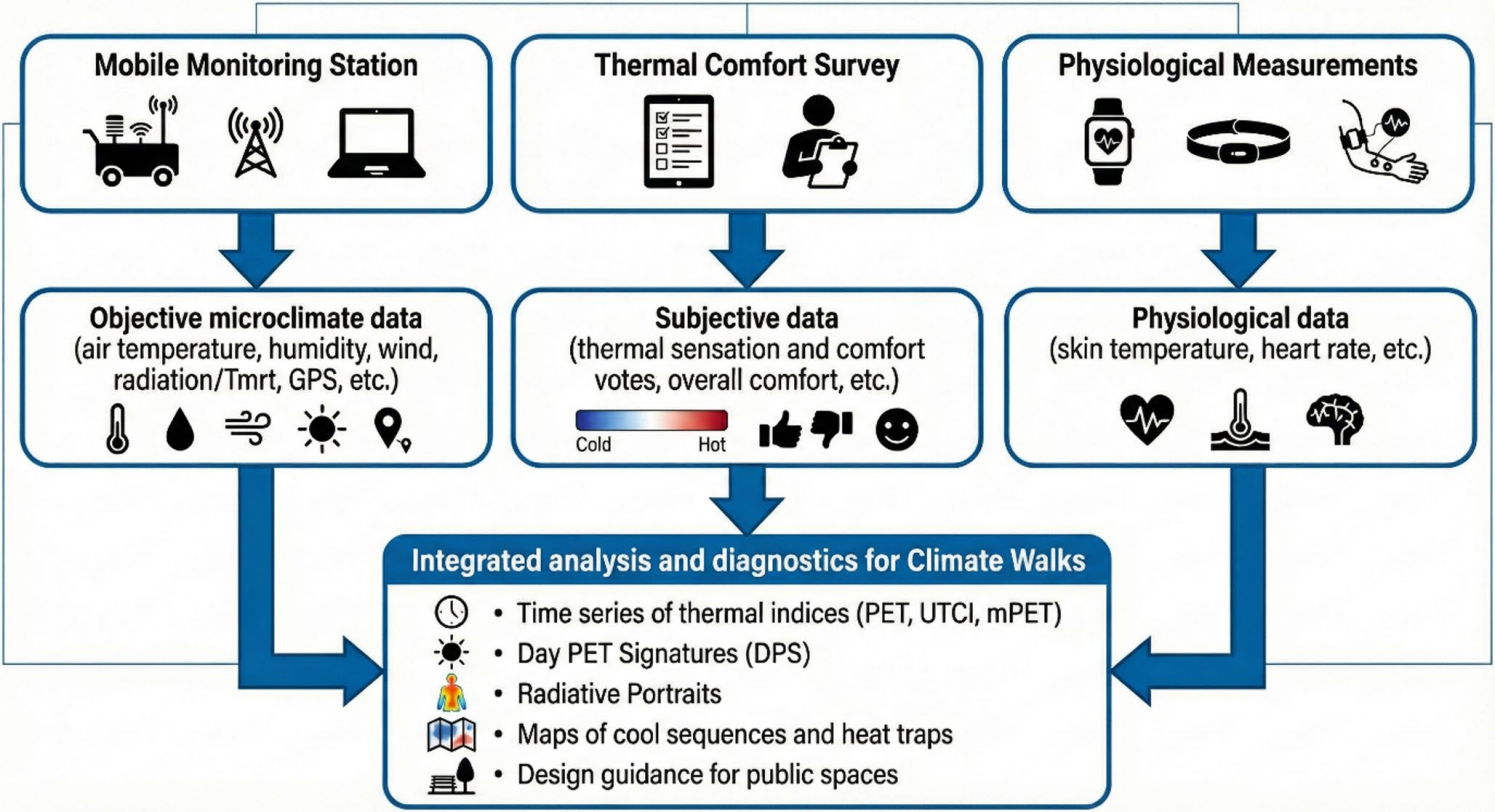
Key components of Climate Walk

We use the term “climate walks” for structured, route-based, georeferenced field assessments that pair mobile microclimate measurements (e.g., air temperature, humidity, wind, radiation/T_mrt_, often summarized by PET/UTCI) with synchronous human responses as participants traverse a variety of urban settings (Fig. 1, Peng et al. 2022; Vasilikou and Nikolopoulou 2020). The approach targets the fine-scale spatial heterogeneity and temporal variability that pedestrians actually encounter, delivering mapped sequences of exposure and perception along a walk. Early formulations drew on georeferenced backpack/wearable setups and stop-and-go protocols developed to monitor transient comfort in complex morphologies (Chokhachian et al. 2018); recent work has broadened access via standardized low-cost, multi-domain rigs such as Portable Low-cost Environmental Monitoring System, PLEMS (Krüger et al. 2024) and introduced richer diagnostics to characterize within-day dynamics and radiative drivers at pedestrian level (Siret et al. 2025).

The emphasis on “walks” reflects the dynamic nature of outdoor thermal experience. As people move between sun–shade and wind regimes, thermal sensation exhibits lags and overshoots (Huang et al. 2025) and is influenced by short-term thermal history (Jiang et al. 2024). Consequently, point-in-time, steady-state assessments underrepresent real behaviour (Höppe 2002). Climate walks are expressly designed around this transience, using stop-and-go timing to align exposure and perception, and employing sequence-oriented information to reveal how place sequences modulate comfort in motion. They also offer complementary data and information to fixed monitoring network by resolving micro-scale heterogeneity and experience in motion for the quantification of human thermal comfort conditions and variability.

### Key characteristics of climate walks

Climate walks are designed to follow the time course of exposure and perception as pedestrians move through alternating sun-shade and wind regimes (Deng et al. 2023; Gallacher and Boehnke 2025), often using stop-and-go timing to separate walking from resting states and to align stimulus with response around transitions (Kim and Kim 2024; Lam et al. 2025). In existing studies, stop phases are typically on the order of one to three minutes, which is generally sufficient for sensors to stabilise and for participants to register the new conditions without unduly interrupting the experience of a continuous walk. Systematic evaluation of optimal stop duration for different climates, instruments and research questions remains a methodological gap and is an important priority for future work on standardised protocols. Field studies consistently document short lags and occasional overshoots in thermal sensation, with sensitivity to the preceding minutes of exposure, patterns that steady-state assessments underrepresent (Lau et al. 2019). Accordingly, climate walks report both constituent meteorology and integrative indices (e.g., PET, UTCI) while remaining index-aware: in hot-humid or radiatively complex settings, and especially during walking, a modified version of PET (mPET) can outperform PET in estimating thermo-physiological responses, warranting sensitivity analyses and dual reporting (Ouyang et al. 2025). Since outdoor experience is multisensory, many climate walks intentionally extend beyond the strictly thermal domain and record additional parameters such as noise, air quality and illuminance; we refer to these as multisensory climate walks, in contrast to thermal walks that monitor only thermal variables and thermal votes. Empirical work on greenways shows acoustic–thermal coupling in which subjective noise loudness and sound level can rival or exceed thermal factors in shaping overall satisfaction (Li et al. 2025), yielding practical ranges for design.

Protocols co-locate microclimate measurements with mapped routes and synchronous subjective votes (and, where feasible, thermos-physiological signals), providing pedestrian-scale resolution at walking pace. Subjective responses are typically collected using established thermal sensation and comfort scales, for example the ASHRAE seven-point category scale or scales based on ISO 10,551. Early georeferenced backpack/wearable setups established the template for coupling measurements to route geometry (Chokhachian et al. 2018); recent implementations add six-directional radiation sensing and rigorous timing to resolve transitions (Requena-Ruiz et al. 2025). Beyond research-grade carts and stations, low-cost backpack systems make climate walks feasible for short circuits and community campaigns without sacrificing multi-domain coverage. The PLEMS platform, for example, integrates thermal, air-quality, light and noise sensors on a microcontroller-based backpack; in repeated “environmental walks” (Krüger et al. 2024), it captured subtle between-position differences and produced subjective-objective consistency at the pedestrian scale. When integrated low-cost platforms such as PLEMS are used, they should be co located with reference grade instruments before and after field campaigns so that any systematic bias or drift can be quantified and, where appropriate, corrected, thereby improving the reliability of the resulting data. Complementary studies compare low-cost devices against reference backpacks to document accuracy and handling trade-offs, that is key information for reproducibility and for scaling citizen-science extensions (Deng et al. 2025; Silva et al. 2025).

Existing climate walk campaigns draw on three broad families of monitoring configurations. Research grade mobile stations, for example trolley or backpack-based systems, are assembled from laboratory sensors and loggers and provide high accuracy at the cost of greater weight, complexity and expense. Integrated low-cost backpacks combine compact sensors and data logging on a single platform, which considerably lowers cost and simplifies deployment while still enabling multi domain monitoring at pedestrian height. Wearable systems, often paired with smartphones, further reduce participant burden and can incorporate physiological monitoring, but their environmental measurements are more sensitive to body interference and shielding. Each configuration involves a different balance between cost, logistical effort, spatial coverage and measurement uncertainty. Across these options, a core set of microclimatic measurements is still required that satisfies recognised outdoor comfort measurement standards and supports the calculation of the selected thermal indices, with additional multisensory modules added as research questions and resources permit.

Climate walks are routed to traverse typologies that structure exposure, including street canyons, squares, arcades, tree rows, waterfronts, and to sample across the day, with transects planned to balance key environmental drivers (Lam et al. 2025) so that shade geometry, sky view, surface materials and façade orientation can be linked to pedestrian-level stress (Smail et al., 2024; Lam et al. 2024). New diagnostics make these dynamics legible: Day PET Signatures (DPS) cluster positions by their diurnal PET paths, and Radiative Portraits decompose short- and long-wave fluxes by direction to reveal how specific radiative configurations drive thermal diversity (Siret et al. 2025). Mapping microclimate across redesigned districts distinguishes persistently exposed “heat-trap” zones from sequences that remain comfortable through the afternoon, while surface-temperature contrasts explain why morphologies with comparable air temperatures can feel markedly different (Jato-Espino et al. 2025; Žgela et al. 2024). In parallel, stop-and-go mobile campaigns show that fine-scale contrasts captured in motion translate into cool-spot identification and timing windows for relief (Lau et al. 2019; Qi et al. 2021)—actionable evidence for targeted shade, vegetation, material choices, and programming of public space (Nouri and Costa 2017).

## Opportunities, limitations, and challenges

Climate walks make it possible to detect and design for thermal diversity at the true scale of pedestrian experience. In the context of more frequent, longer, and more intense heat waves, they provide rapid, place-specific evidence to prioritise heat-mitigation and management at the pedestrian scale (Abuwaer et al. 2025). By following sun–shade transitions, short bursts of wind and evapotranspiration patterns while collecting co-located votes, they reveal time windows and micro-sites of relief for thermal pleasure that steady, point measurements cannot capture (Peng et al. 2022). Recent diagnostics summarize within-day comfort dynamics and tie them to radiative drivers from façades, sky view, trees, and materials, yielding maps of “cool spots” and “heat traps” that are legible to planners and designers (Dzyuban et al. 2022; Parison et al. 2023). In transient field experiments, thermal sensation can overshoot after a change and then adapt within minutes, underscoring why route-based methods are well-suited to identify brief, designable moments of comfort (Jiang et al. 2024; Lau et al. 2019).

Climate walks also support rapid, before/after assessment of interventions, for example, comparing spaces or blocks with differing morphology or greening, to evaluate benefits at the pedestrian scale (Lam et al. 2024; Parison et al. 2023). Beyond thermal exposure, climate walks can be implemented as multisensory climate walks that explicitly target multidomain comfort (Grapas et al. 2025; Kavee and Flanigan 2025): autumn greenway studies, for example, show that subjective noise loudness can outweigh temperature for overall satisfaction, suggesting actionable ranges for acoustic and thermal parameters (Cureau et al. 2022; Krüger et al. 2024). These insights translate directly into design moves (shade/greening placement, façade articulation, water and sound-masking features) and programming aligned to diurnal comfort windows.

Transients and thermal memory of pedestrians pose substantial challenges as they tend to integrate the last few minutes of exposure, producing lags and overshoots in sensation; protocols therefore need explicit integration windows and to distinguish walking from resting states (Vasilikou and Nikolopoulou 2020). In practice, we recommend that subjective votes are interpreted in relation to a defined exposure window, for example the preceding two to three minutes, and that studies report whether participants were walking or resting during that window.

Second, studies vary in route length, pace, stop duration, sensor height/placement, sampling rates, vote intervals and survey language for subjective scales (Schweiker et al. 2020). To improve reproducibility and enable cross study synthesis, we recommend that climate walk campaigns at minimum document and report route geometry and pace, stop length, sensor mounting height and shielding, sampling and vote intervals, clothing and activity, and the exact wording and language of subjective scales, in line with recent calls for harmonised practice (Barbano et al. 2024; Qi et al. 2021; Silva et al. 2025). To support cross study comparability, climate walk campaigns should, as far as possible, adopt established thermal sensation and comfort scales and report their exact wording and anchors. Agreeing on a common set of recommended scales for different climate walk applications is an important task for the next phase of work on standardised protocols.

Third, mobile measurements of radiation and airflow are error-prone; careful protocols (warm-up/initialization, transition data exclusion, outlier handling) and standardised practice for T_mrt_ estimation and instrument calibration are essential, especially with low-cost rigs (Barbano et al. 2024; Krüger et al. 2024). Capturing rapid transition events stresses sensor response and shielding; this remains a known limitation and priority for validation, warranting reported time constants, sync accuracy, and post-transition exclusion windows. Comparative studies of mobile stations indicate that such low cost systems can provide useful data when they are co-located with a trusted reference station before and after campaigns (Gallacher et al. 2024; Xu et al. 2025), when simple bias corrections are derived and applied, and when basic performance specifications, including accuracy, response time and shielding conditions, are documented. As the field moves towards standardisation, aligning minimum requirements for these specifications with existing outdoor comfort measurement standards will be important for comparability between studies. Thermal indices can also rank stress differently in hot-humid or radiatively complex settings (Kim and Kim 2024; Ouyang et al. 2025), so sensitivity analyses are advisable when pedestrians move between sun and shade.

Fourth, confounding multisensory effects may play an important role in subjective thermal perception, such as acoustics interacting with thermal perception. Ignoring noise (or other senses) may also risk misattributing discomfort to “heat” and misguiding design (Cureau et al. 2022). To reduce this risk, we recommend that campaigns which aim to infer thermal satisfaction at least document ambient noise conditions and, where feasible, include simple questions on noise, light and overall comfort.

Lastly, many campaigns concentrate on one season, in fair weather conditions or during limited hours (e.g., autumn greenways, summer residential walks), leading to issues in sampling representativeness. Broader coverage across seasons, weather types, and times of day, including crowding and activity rhythms, improves generalisability of findings. Where such coverage is not feasible, we recommend that authors describe the sampling frame explicitly and frame design implications as conditional on the sampled conditions.

Looking ahead, experience from existing climate walk campaigns suggests a common sequence of stages that can guide future work. These stages include clarifying study aims and policy or design context, selecting routes and periods that are meaningful for the target population and climatically diverse, choosing and configuring a core set of microclimate instruments together with GPS and any physiological sensors, defining participant inclusion criteria and ethical procedures, harmonising protocol elements such as walking pace, stop locations and vote timing, and specifying how environmental, physiological and questionnaire data streams will be aligned and analysed. We offer this sequence as an initial sketch toward standardised procedures, and see the co-development of a more detailed framework and graphical flowchart as a priority for the next phase of research.

## Future research directions

Table 1 summarises the future research direction of climate walk. A central research need is turning thermal pleasure under dynamic conditions into design rules for outdoor and semi-outdoor routes. This means mapping and intentionally sequencing short sun–shade–breeze alternations that deliver “micro-relief” without overshooting into discomfort (Lau et al. 2019; Requena-Ruiz et al. 2025), and validating them with time-resolved diagnostics along actual walks (Peng et al. 2022). Evidence from dynamic comfort studies and thermal-walk campaigns shows lagged/overshoot responses, implying designs should target contrast magnitude and timing, not only absolute set-points (Dzyuban et al. 2022; Jiang et al. 2024). Multisensory models should be coupled in, since sensory stimuli and thermal appraisals interact in motion, warranting cross-city tests across walking/resting states and seasons (Lam et al. 2024).

**Table 1:** Future research direction of climate walks and design implications

Theme	Research gap	Significance	Future directions
Dynamic thermal pleasure to inform design rules	No actionable rules that translate sun–shade–breeze alternations (and lag/overshoot effects) into route design.	People perceive *changes* (contrast & timing), not just steady set-points; designs risk overshooting comfort.	Map and intentionally sequence short sun–shade–breeze “micro-relief” segments; optimize contrast magnitude & timing; validate with time-resolved, along-walk diagnostics.
Multisensory comfort in motion	Weak coupling of thermal with other sensory stimuli; limited cross-city/seasonal, walk vs. rest coverage.	Comfort judgments on the move are multisensory; single-city or single-season results don’t generalize.	Couple multisensory models with thermal appraisal; run cross-city, cross-season tests across walking/resting states.
Time-resolved, memory-aware metrics	Point estimates/steady indices ignore ~ 2–3 min sensation integration during movement.	Misrepresents exposure and transitions that drive votes/physiology.	Develop time-resolved, memory-aware exposure summaries using ~ 2–3 min integration windows; compare against subjective/physiological responses.
Index performance under motion	Thermal indices rarely validated for walking; index choice shifts stress classes in complex climates.	Wrong index, therefore wrong risk/design decisions.	Systematically test indices in motion (e.g., mPET vs. PET) under hot-humid & radiatively complex settings; publish confusion/error analyses and guidance.
Dynamic diagnostics & instrumentation	Limited capture of directional radiative and ventilation transients; weak linkage to indices.	Misses the transitions that create relief or stress along routes.	Pair DPS with radiative portraits (and prospective wind portraits); align with thermal indices to interpret transitions and compare sites.
Harmonized protocols & reporting	Inconsistent reporting hinders replication and cross-site synthesis.	Without standards, findings aren’t comparable across hours, seasons, or cities.	Adopt a concise reporting checklist: route geometry & pace; stop length; sensor specs/placement & Tmrt method; survey questions; sampling/vote intervals; clothing & activity; weather class; plus data/metadata sharing.
From points to sequences (design practice)	Focus on isolated “cool spots” instead of connected, route-scale choreography aligned to sun/wind.	Users traverse networks; relief must persist across time and space.	Choreograph sequences robust to hour-of-day, route, and use; use DPS clustering to flag time-dependent heat traps vs. protected positions; use radiative portraits to design directional shielding.
Site specificity & validation in motion	Large context-dependence of cool-spot benefits; one-size-fits-all rules underperform.	Avoid overgeneralized prescriptions that fail locally.	Validate in motion on site; build typologies of street canyons / trees / arcades and decision trees for sequence design per context.
Operationalizing climate walks	The bridge from biometeorology to actionable public-space design is still thin.	Needed to inform climate-resilient, health-promoting streets.	Use georeferenced, dynamic, human-centred climate walks as a standard assessment; package design-relevant outputs (maps, segments, sequences) for planners; align with climate-change adaptation.

Comfort metrics should move beyond point estimates toward time-resolved, memory-aware exposure summaries that reflect the 2-3-min integration of human sensation during movement (Deng et al. 2025; Vasilikou and Nikolopoulou 2020). In parallel, index performance under motion must be tested systematically; recent walking experiments show mPET Lin et al. (2019) outperforming PET against measured physiological factors, indicating that index choice can change stress classification in hot-humid or radiatively complex settings (Ouyang et al. 2025). Pairing DPS with radiative (and prospective wind) portraits can also provide dynamic diagnostics to capture directional radiative and ventilation transients, and associate with thermal indices to interpret transitions and compare across sites. These needs argue for a concise reporting checklist, including route geometry/pace, stop length, sensor specs/placement and T_mrt_ method, survey questions, sampling/vote intervals, clothing/activity, and weather class, in order to enable comparability across sites and seasons, building on stop-and-go “climate walk” practice.

Design should shift from locating single refuges to choreographing connected sequences that align with daily sun paths and prevailing winds and remain effective across hours, routes, and uses (Requena-Ruiz et al. 2025). DPS clustering already distinguishes time-dependent “heat traps” from reliably protected positions, while radiative portraits expose the directional radiative configurations that make sequences work (Siret et al. 2025). Mobile studies of cool spots further show large radiative/T_mrt_ and subsequent PET reductions but with strong context-dependence (Parison et al. 2023), underscoring why sequence design must be site-specific and validated in motion rather than guided by one-size-fits-all rules. In practical planning applications, these diagnostics can be translated into actionable outputs such as route based thermal comfort maps that identify cool sequences and heat traps, or temporal shade availability charts that reveal how radiative exposure evolves along key pedestrian routes across the day or season. Such products help planners and designers assess existing conditions and prioritise microclimate sensitive interventions at the spatial and temporal scales actually experienced by pedestrians.

In this paper, we identify the nexus between human thermal comfort and urban microclimate, clarify knowledge gaps in micro(bio)climate monitoring, and define climate walks as georeferenced, dynamic, human-centred assessments of pedestrian scale thermal experience. We synthesize their key characteristics, methodological trade-offs, and design relevant outputs to outline an agenda that operationalizes dynamic thermal pleasure and multisensory comfort, develops time resolved metrics and tests index performance under motion within harmonized protocols, and shifts practice from isolated cool spots to connected, route scale cool sequences. A key next step will be the collective development of shared procedural frameworks and flowcharts for climate walks, translating the initial sequence sketched here into more detailed, consensus-based standards that can support comparable, practice ready evidence across contexts. Collectively, these contributions and the call for coordinated methodological development are intended to link biometeorology to actionable public space design under climate change.

## Supplementary Information

Below is the link to the electronic supplementary material.

Supplementary Material 1.

## Data Availability

No datasets were generated or analysed for this short communication. The article is based on existing, published literature; therefore, no data are available.
