# Supplementary material for: From street level to science: advancing methods for climate walks to improve human thermal comfort
Source: Int J Biometeorol. 2026 Apr 2;70(4):112. doi: 10.1007/s00484-026-03167-8 (PMC13046601; doi:10.1007/s00484-026-03167-8)
Supplement: Supplementary file 1 — Supplementary Material 1. [file 484_2026_3167_MOESM1_ESM.docx]

**Supplementary Materials**

***Table S1.*** *Comparison of instruments employed in studies covered by the present study.*

| **Instrument/Logger** | **Parameters Measured** | **Sensor Type** | **Accuracy** | **Sampling/Response Time** | **Mounting Type** | **Source** |
| --- | --- | --- | --- | --- | --- | --- |
| TESTO 480 / 440 | Air temperature (T_a_), Relative humidity (RH), Wind speed (v), Globe temperature (T_g_), Surface temperature (T_s_) | Vane probe, Humidity/temp probe, Waterproof surface probe, Type K thermocouple globe (150 mm) | T_a_: ± 0.2 °C; RH: ± 1.0%; v: ± 0.03 m/s; T_g_: ± 1 °C | 5 s steps or 1 min intervals | Backpack or Tripod (1.4–1.7 m) | Ali Smail et al., 2024  Chokhachian et al., 2018  Kim & Kim, 2024  Kim et al., 2022 |
| Kestrel 5400 Heat Stress Tracker | T_a_, RH, v, T_g_, UTCI | Integrated meteorological sensors | Ta: < ± 0.5 °C; RH: < ± 2%; v: < ± 3%; T_g_: < ± 1.4 °C | 2 s to 5 s steps | Backpack or Foldable tripod | Deng et al., 2023  Lam et al., 2024  Peng et al., 2022 |
| Trolley-based Environmental Monitoring System (TEMS) | T_a_, T_g_, RH, v, PM, T_s_, GPS, SVF | Type K thermocouple, HOBO MX1101, Graywolf PC-3500, Kimo CTV110, Flir T335 | T_a_: ± 0.21 °C; RH: ± 2%; v: ± 3%; T_s_: ± 2 °C | 1 s (Datalogger); 1 min (PM) | Trolley (sack truck) | Lam et al., 2025 |
| MWS 1 & 2 | T_a_, T_g_, v, RH, Radiation (SW/LW), GPS, Atmospheric pressure (P), PM, sound, UTCI | Tinkerforge and Meter sensors; Gills Maximet GMX500, Apogee SN500, Campbell globe | T_a_: ± 0.1 to 0.3 °C; RH: ± 2%; v: 0.3 to 1.1 m/s; T_g_: ± 0.3 to 0.7 °C | 5 s | Backpack or All-terrain cart | Silva et al., 2025 |
| Pedestrian-carried / Mobile station | T_a_, RH, v, Radiation (SW/LW) | Vaisala HMP155A, Gill 2D WindSonic, Hukseflux NR01 (x3) | T_a_: ± 0.2 °C; v: ± 2%; Radiation: ± 10% | 0.5 Hz sampling; 18–20 s response | Modified bicycle trailer or climate cart | Requena-Ruiz et al., 2025  Siret et al., 2025 |
| PLEMS (ESP32-based) | T_a_, RH, T_g_, v, CO_2_, Illuminance, Sound pressure, GPS | Multi-sensor (AHT10, DS18B20, ANBR-1, MQ-135, BH1750-FVI, KY-038) | T_a_: ± 0.3 °C; RH: ± 2%; T_g_: ± 0.5 °C; v: < ± 5%; CO_2_: ± 5% | 60 s cycle | Backpack | Krüger et al., 2024 |
| TRM-ZS2-BX Weather Station | T_a_, RH, v, Solar radiation (G), T_g_ | PTS-3, EC-A2 ultrasonic, TBB-2 net radiation, PTWD-2A globe | T_a_: ± 0.2 °C; RH: ± 2%; v: ± 2%; G: ± 1 W/m^2^; T_g_: ± 0.2 °C | 1 min intervals | Stationary sidewalk (1.5 m) | Huang et al., 2025 |
| Delta OHM HD 32.2 / 32.3 | T_a_, RH, v, T_g_, Sound level | Multi-probe (50 mm globe); SMART SENSOR AS-824 | T_a_: ± 0.21 °C; RH: ± 2.5%; v: 0.1 m/s resolution | 15 s to 1 min | Backpack or Hand-carried | Li et al., 2025  Qi et al., 2021  Speak & Salbitano, 2022 |
| MeteoTracker (IoTopon Srl) | T_a_, RH, P | Capacitive–resistive sensors | T_a_: ± 0.5 °C; RH: ± 2%; P: ± 50 Pa | $\geq$ 1 Hz | Magnetic base or String | Barbano et al., 2024 |
| Davis Vantage Pro 2 Plus | T_a_, RH, v, G | Silicon diode, Film capacitor, Solid state magnetic sensor | T_a_: ± 0.3 °C; RH: ± 2%; v/G: ± 5% | 1 min steps | Wheeled tripods | Kim et al., 2022 |
| Atmos 41 | T_a_, RH, v, G | Integrated weather station | T_a_: ± 0.6 °C; RH: ± 1.5%; v: ± 3%; G: ± 5% | 1 min | Wheeled tripods (1.1 m) | Kim & Kim, 2024 |
| LI-COR LI-1500 | G, GPS tagging | Pyranometer LI-200R, RADIONOVA RF Antenna | G: 0.183 W/m^2^; GPS: 2.5 m | 5 s steps | Backpack | Chokhachian et al., 2018 |

***Table S1 (continued).*** *Comparison of instruments employed in studies covered by the present study.*

| Instrument/Logger | Parameters Measured | Sensor Type | Accuracy | Sampling/Response Time | Mounting Type | Source |
| --- | --- | --- | --- | --- | --- | --- |
| MS1 & MS2 (Mobile Stations) | T_a_, RH, v, T_g_, G | Delta OHM HD32.3, HD2102.2, HD52.3D17 | Not in source | Not in source | Backpack or Cart | Deng et al., 2025 |
| HOBO U23 Pro v2 / U12-014 | T_a_, RH, T_g_ | Type-T Copper-Constantan thermocouple (40 mm ball) | T_a_: ± 0.2 °C; T_g_: ± 1.5 °C | 1 min planned stops | Walking tripod (1.6 m) | Chàfer et al., 2022 |
| Wearable system / Station | T_a_, CO_2_, RH, v, P, G, Illuminance, PM | SHT35, TDS0037 (NDIR), calibrated sensors | T_a_: ± 0.1 °C; CO_2_: ± 2% | t_90_ < 8 to 30 s | Wearable or Backpack | Cureau et al., 2022  Grapas et al., 2025 |
| Multi-parametric station | T_a_, RH, v | Not in source | T_a_: ± 0.3 °C; RH: ± 2%; v: ± 3% | 10 s | Van | Grapas et al., 2025 |
| Helmet/Backpack Sensors | T_a_, RH, P, G, v, CO_2_, CO, VOC, T_s_ | BME280, SP-215, CV7-OEM, DYNAMENT MSH-P-CO2, FLIR vue Pro R | T_a_: ± 0.5 °C; v: 0.13 m/s; CO_2_: ± 2%; T_s_: ± 5 °C | 1 s to 30 s (t_90_) | Helmet or Backpack | Pigliautile & Pisello, 2018 |
| Stop-and-go station (Parison 2023) | T_a_, RH, T_g_, v | Sheltered Pt100, Capacitive hygrometer, Hot-wire anemometer | T_a_: ± 0.1 °C; RH: ± 1.5%; T_g_: ± 0.15 °C | 10–20 min stabilization | Mobile station | Parison et al., 2023 |
| MaRTy (mobile platform) | T_a_, RH, Radiation (SW/LW), v | Six-direction sensors | Not in source | Not in source | Human-biometeorological cart | Dzyuban et al., 2022 |
| MoBio Platform | T_a_, RH, v, G, Fluxes | Integrated (Hukseflux, Gill, Vaisala) | Not in source | 2 s intervals | Pedestrian-carried / Push-cart | Ouyang et al., 2025 |
| HR-500 | PM1, PM2.5, PM10, CO_2_ | Not in source | PM: 0.1 g/m^3^; CO_2_: 1 ppmv | 1 s | Backpack | Lam et al., 2024 |
| TES 1339R lux meter | Illuminance (lx) | Lux meter | ± 3% of readings | 1 s | Backpack | Deng et al., 2023  Lam et al., 2024 |
| IoT MF-300 | T_a_, GPS | Temperature probe | T_a_: ± 0.2 °C; GPS: 3.0 m | 1 s | Backpack (cycling) | Žgela et al., 2024 |
| Custom-made bike station | T_a_, T_s_, RH | HD 500 thermologger | Not in source | 1 measurement/s | Bicycle | Jato-Espino et al., 2025 |
| Fluke TI400 | T_s_ | Thermal imaging camera | Range -20 to 80 °C | Site-specific scans | Handheld | Jato-Espino et al., 2025 |
| Backpack station (helper) | T_a_, RH, v, T_g_ | Tailor-made globe (38 mm); Thermocouple wire | T_a_: ± 0.2 °C; RH: ± 1.0%; v: ± 0.03 m/s; T_g_: ± 1.1 °C | 1 s interval | Backpack | Lau et al., 2019 |
| Self-built device | T_a_, RH, P, T_s_, G, T_g_ | Pyranometer; GNSS | VDI 2023 standards | 1 s recording | Pedestrian (GoPro stick) | Gallacher & Boehnke, 2025 |
| Campbell Scientific CR800 | T_a_, RH, T_g_, v, Illuminance | CS215, CT100, Ultrasonic 2D, Skye lux meter | Not in source | Not in source | Aluminum trolley (1.75 m) | Vasilikou & Nikolopoulou, 2020 |
| Gill GMX501 System | T_a_, RH, v, G | Sonic anemometer, CNR4 net-radiometer, Apogee thermistor | Not in source | 15 s to 5 min | Stationary tripod or Cart | Aleksandrowicz & Pearlmutter, 2023 |
| Sound Level Meters | Sound pressure level / Decibel | AS-K8; C.A. 832 | ± 1.5 dB | 1 s | Backpack | Cureau et al., 2022  Lam et al., 2024 |
